# POSTOPERATIVE STABILITY AFTER VOLAR LOCKING PLATE IN DISTAL RADIUS FRACTURES

**DOI:** 10.1590/1413-785220263403e300613

**Published:** 2026-06-12

**Authors:** Luiz Henrique Santos Cambrussi, Douglas Barbosa Lima, Marcos Afonso Grando, Renata Mello Calandrini, Bruno Adona Ribeiro

**Affiliations:** 1 Hospital Universitario da Universidade Estadual de Ponta Grossa, Ponta Grossa, Parana, Brazil.

**Keywords:** Internal Fixators, Fracture, Distal Radius, Radiographic Image Interpretation, Computer-Assisted, Bone Plates, Fixadores Internos, Fraturas Distais do Rádio, Interpretação de Imagem Radiográfica Assistida por Computador, Placas Ósseas

## Abstract

**Objective:**

To evaluate the correction and maintenance of radiographic parameters—volar tilt (VT), radial inclination (RI), and radial height (RH)—from the preoperative period to outpatient discharge in distal radius fractures treated with a volar locking plate (VLP), stratified by AO/OTA, Fernandez, and IDEAL classifications.

**Methods:**

Retrospective analytical case series including 135 fractures operated on between January 2023 and August 2024. Standard anteroposterior and lateral radiographs were obtained at three time points: preoperative (PRE), immediate postoperative (IPO), and outpatient discharge (POT). Two independent, mutually blinded raters classified the fractures and measured VT, RI, and RH. The study focused on radiographic outcomes.

**Results:**

Significant improvements in VT, RI, and RH were observed from PRE→IPO and PRE→POT (p<0.001). Differences from IPO→POT were small and not significant (p>0.05), indicating maintenance of parameters until discharge. The same pattern was seen in higher-complexity subgroups (AO type C, IDEAL 3, and Fernandez 5).

**Conclusion:**

VLP fixation provided effective initial correction and radiographic maintenance up to discharge in distal radius fractures, particularly in complex patterns. ***Level of evidence IV, case series.***

## RESUMO

**Objetivo:**

Avaliar a correção e a manutenção dos parâmetros radiográficos — inclinação volar (IV), inclinação radial (IR) e altura radial (AR) — do pré-operatório à alta ambulatorial em fraturas distais do rádio tratadas com placa volar bloqueada (PVB), estratificando pelas classificações AO/OTA, Fernandez e IDEAL.

**Métodos:**

Estudo tipo série de casos retrospectiva analítica com 135 fraturas operadas entre janeiro/2023 e agosto/2024. Radiografias em anteroposterior e perfil nos momentos pré-operatório (PRE), pós-operatório imediato (POI) e alta (POT). Dois avaliadores independentes, cegos entre si, classificaram as fraturas e mensuraram IV, IR e AR. O estudo concentrou-se em desfechos radiográficos.

**Resultados:**

Houve correção significativa de IV, IR e AR entre PRE→POI e PRE→POT (p<0,001). Entre POI→POT, as diferenças foram pequenas e não significativas (p>0,05), indicando manutenção dos parâmetros até a alta. O padrão repetiu-se nos subgrupos de maior complexidade (AO C, IDEAL 3 e Fernandez 5).

**Conclusão:**

A placa volar bloqueada proporcionou correção inicial eficaz e manutenção radiográfica até a alta nas fraturas distais do rádio, sobretudo em fraturas complexas. ***Nível de evidência IV; série de casos.***

## INTRODUCTION

Distal radius fractures are common and account for about 15% of all fractures in adults, with a bimodal distribution: in the elderly, due to bone fragility, and in young adults, due to high-energy trauma^1,2^.

Standardized radiographic evaluation — radial inclination (RI), radial height (RH), and volar tilt (VT) — is crucial for guiding treatment and follow-up. Reference values (RI ~23°, RH ~11–12 mm, VT ~11°)^3^ are often used as postoperative goals, although they vary according to sex, age, and individual characteristics. It is important to emphasize that the relationship between radiographic alignment and function is not linear^4^.

Among surgical options, the volar locking plate (VLP) stands out for providing stable angular fixation and allowing early mobilization, with favorable radiographic results even in more complex fractures^5^. However, complications and late loss of reduction may occur, which reinforces the importance of monitoring the maintenance of parameters throughout follow-up — and not just immediate correction^6^.

The morphological and mechanical heterogeneity of fractures requires complementary classification systems. The AO/OTA organizes fractures by morphology^7^; Fernandez focuses on the mechanism of injury^8^; and the IDEAL — a Brazilian proposal — integrates aspects of the patient, trauma, and fracture (Instability, Deviation, Joint extension, Association, and Soft tissue injury)^9^, offering an expanded view of severity and instability. The interobserver reliability of classifications based solely on X-rays is usually moderate, but it can improve with computed tomography (CT) in intra-articular fractures; nonetheless, routine clinical application must balance accuracy and availability.

Despite the robust literature on initial outcomes VLP, the maintenance of RI, RH, and VT from the immediate postoperative period to outpatient discharge remains underexplored, especially in complex patterns.

The aim of the study is to investigate the correction and stability of radiographic parameters over the postoperative follow-up, stratifying by AO/OTA, Fernandez, and IDEAL, and to verify whether these measures are maintained until outpatient discharge with the use of a locked volar plate, including in fractures of greater complexity.

## METHODOLOGY

Analytical retrospective case series study, approved by the Research Ethics Committee (CAAE: 84384024.5.0000.0105).

All cases with a diagnosis of distal radius fracture treated with VLP and aged over 18 years, operated between January 2023 and August 2024 at a tertiary reference hospital in orthopedics in the southern region of Brazil, were included.

Cases with absence of preoperative X-rays or loss of outpatient follow-up were excluded.

The X-rays were standardized in anteroposterior and lateral views at three time points: preoperative (PRE), immediate postoperative (POI), and outpatient discharge (POT).

The surgical indication with locked volar plating followed the institutional protocol for unstable and/or articular fractures: articular displacement >2 mm, volar/dorsal angulation >10°, loss of radial height >3 mm, metaphyseal comminution, and/or articular involvement. All procedures were performed by the same hand surgeon, using the same plate model, via Henry's volar approach.

Fractures were classified by two independent evaluators (one hand surgeon and one third-year resident in orthopedics and traumatology) using the AO/OTA, Fernandez, and IDEAL systems. Discrepancies were resolved by consensus.

For radiographic evaluation, the parameters used were: volar tilt (VT), radial height (RH), and radial inclination (RI). Measurements were made in the Animati PACS system at three time points: PRE (before the surgical procedure), POI (immediate postoperative), and POT (at the time of outpatient discharge), with the evaluators blinded to each other's measurements.

The intraclass correlation coefficient (ICC) was estimated for VT, RH, and RI, interpreted by ranges (low <0.50; moderate 0.50–0.75; good 0.75–0.90; excellent >0.90).

The normality of the distributions was assessed using the Shapiro–Wilk test. Comparisons between the three time points were performed using the Friedman test, with Nemenyi post-hoc test for multiple comparisons. In the descriptive analysis, means, medians, standard deviation (SD), and quartiles (P25–P75) were reported. Significance level α = 0.05. Exploratory study, without prior power calculation. The analyses were conducted in R 4.1.3 (R Core Team, 2022). Informed consent was waived as this was a retrospective review of records, without nominal identification.

## RESULTS

A total of 135 fractures were analyzed. The sample was predominantly composed of female patients (76 women and 59 men), and the majority (63.7%) were over 50 years old. A predominance of complex patterns was observed in the three classifications: AO type C = 69.63% (A = 17.78%; B = 12.59%), IDEAL 3 = 52.59% (IDEAL 2 = 47.41%) and Fernandez 3/5 = 35.56% each (Table 1).

**Table 1:** Frequency of fracture types according to each classification system.

				CI (95%)	
Variable		N	%	Inf	Sup
AO	A	24	17.78%	12.25%	25.09%
	B	17	12.59%	8.01%	19.24%
	C	94	69.63%	61.42%	76.75%
Ideal	2	64	47.41%	39.17%	55.79%
	3	71	52.59%	44.21%	60.83%
Fernandez	1	23	17.04%	11.63%	24.27%
	2	16	11.85%	7.43%	18.39%
	3	48	35.56%	27.98%	43.93%
	5	48	35.56%	27.98%	43.93%

N= absolute frequency; = relative frequency; Inf= lower limit of the 95% CI; sup= upper limit of the 95% CI. IC: Confidence interval.

In the preoperative phase (PRE), the volar inclination (VI) predominantly showed dorsal values; after the osteosynthesis with a locked volar plate, there was substantial correction in the immediate postoperative phase (POI), with maintenance at the outpatient discharge (POT). Similar patterns were observed for radial inclination (RI) and radial height (RH) (Table 2).

**Table 2:** Radiographic parameters by moment (mean ± SD).

Variable	PRE (n=135)	POI (n=135)	POT (n=134)
Volar inclination (in degrees)	-13.87 ± 18.06	5.65 ± 5.23	5.89 ± 4.73
Radial inclination (in degrees)	12.67 ± 7.15	18.11 ± 4.55	18.62 ± 4.31
Radial height (in millimeters)	4.26 ± 5.73	10.10 ± 3.33	10.03 ± 3.19

PRE= pre-operative; POI= immediate post-operative; POT= outpatient discharge.

The greatest variations occurred between PRE→POI and PRE→POT for VT, RI, and RH (p<0.001). Between POI→POT, the differences were small and not significant (p>0.05) for the three parameters, indicating stability throughout the follow-up (Table 3). Additionally, the comparisons between the differences PRE→POT vs PRE→POI were not significant (VT p=0.371; RI p=0.264; RH p=1.000), while the comparisons involving POI→POT were significant (p<0.001), reinforcing the maintenance of the parameters between POI and POT.

**Table 3:** Descriptive analysis of the differences between the evaluated moments.

Variable	Δ PRE→POI (mean ± SD)	Δ PRE→POT (mean ± SD)	Δ POI→POT (mean ± SD)	Significance (Friedman + Nemenyi)
Volar inclination (in degrees)	19.51 ± 17.79	19.88 ± 17.81	0.22 ± 4.12	PRE vs POI: p<0.001; PRE vs POT: p<0.001; POI vs POT: p> 0.05
Radial inclination (in degrees)	5.44 ± 7.19	6.01 ± 6.84	0.54 ± 3.60	PRE vs POI: p<0.001; PRE vs POT: p<0.001; POI vs POT: p> 0.05
Radial height (in millimeters)	5.83 ± 5.42	5.84 ± 5.35	-0.04 ± 2.01	PRE vs POI: p<0.001; PRE vs POT: p<0.001; POI vs POT: p> 0.05

PRE = pre-operative; POI = immediate post-operative; POT = outpatient discharge; SD = standard deviation.

**Figure 1:**
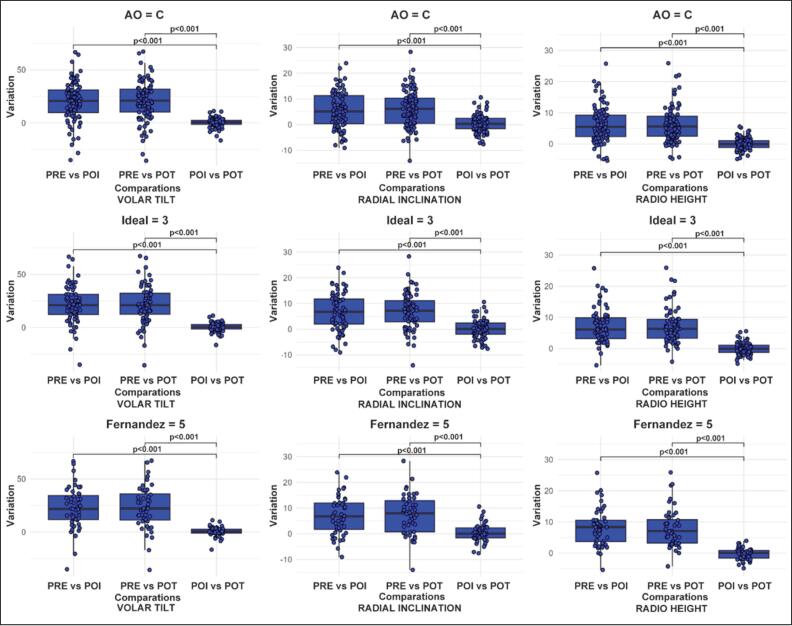
Boxplot of the variation in volar inclination, radial inclination, and radial height between the evaluated times for (AO = C, IDEAL = 3, and FERNANDEZ = 5).

The same pattern — significant initial correction and subsequent maintenance — was observed in the subgroups AO type C, IDEAL 3, and Fernandez 5, with no clinically relevant differences between POI and POT. Figure 1 presents the boxplots of the more complex fractures (AO type C, IDEAL type 3, and Fernandez type 5), highlighting the significant change in the measured parameters between the preoperative phase and the other moments, as well as the maintenance of these measures between the immediate postoperative phase and outpatient discharge.

## DISCUSSION

The findings align with publications that indicate good radiographic results and the biomechanical advantage of the VLP, favoring early mobilization in unstable/comminuted patterns^5,10,11^.

Previous clinical studies describe satisfactory maintenance of alignment after volar fixation, although late variations may occur, especially in the absence of well-positioned subchondral screws^12^. On the other hand, in complex articular fractures, not all parameters always return to physiological ranges by the end of the follow-up^13^, which emphasizes the importance of analyzing subgroups by complexity through fracture stratification using classifications.

The simultaneous use of three systems (AO/OTA, Fernandez, and IDEAL) allowed for discussion of the phenomenon of radiographic maintenance from complementary dimensions (morphology, mechanism, and clinical/radiographic instability).

Furthermore, the predominance of complex patterns (e.g., AO C) is consistent with the tertiary setting and provides a demanding test of implant stability; the fact that complex patterns also demonstrate maintenance between POI and POT suggests that the VLP adequately meets this biomechanical demand.

Although alignment and radiographic parameters have clinical relevance, they are not synonymous with full functional recovery. Part of the literature shows a non-linear correlation between radiographic measures and clinical outcomes (pain, grip strength, range of motion, PRWE/DASH scales)^4^, especially when there is residual dorsal deviation. Our design did not capture functional outcomes; therefore, one should not automatically infer clinical superiority based solely on radiographic stability.

In practice, the data indicate that, in the absence of clinical signs of failure, reducing late radiographic controls may be considered until outpatient discharge, as POI→POT remained stable. This strategy may optimize workflows and reduce costs/radiation exposure while preserving good clinical follow-up.

The numerically significant consecutive sample, standardization of technique/implant/surgeon, reducing operator variation, the double blind measurement of radiographic parameters with reported ICC, and stratification by three classification systems allow for a multidimensional reading of the results.

However, the fact that it has a retrospective design in a single center and with a predominance of complex fractures may restrict generalization. The absence of functional outcomes and clinical and demographic covariates reduces the ability to adjust for confounders and reinforces the descriptive nature of the inferences.

The findings suggest that VLP offers effective correction and maintenance of alignment until outpatient discharge, supporting the use of the method in these standards, provided clinical decisions consider function, symptoms, and the individual context of each patient^4,14^.

Prospective and multicenter studies, with standardized collection of clinical/demographic variables and inclusion of functional outcomes (PRWE, DASH, strength, range of motion), are necessary to confirm these findings over longer horizons and estimate the clinical impact and cost-effectiveness of follow-up protocols with fewer radiographs when clinical evolution is favorable.

## CONCLUSION

Significant correction of VT, RI, and RH was observed from pre-operative to immediate post-operative and maintenance of these parameters until outpatient discharge. This behavior was maintained in the subgroups of greater complexity (AO C, IDEAL 3, and Fernandez 5), suggesting implant stability in the initial follow-up, even in more complex fractures.

VLP presents itself as an effective option for early radiographic correction and maintenance, with therapeutic and follow-up decisions needing to consider individual clinical evolution, symptoms, and patient context. Prospective and multicenter studies, with standardized collection of covariates and inclusion of functional outcomes, are necessary to confirm and expand this evidence.

## Data Availability

The contents underlying the research are available in the manuscript.
